# The Role of Housing Conditions on the Success of Artificial Insemination in Intensively Reared Dairy Ewes in Greece

**DOI:** 10.3390/ani12192693

**Published:** 2022-10-07

**Authors:** Stergios Priskas, Georgios Valergakis, Ioannis Tsakmakidis, Sotiria Vouraki, Vasiliki Papanikolopoulou, Alexandros Theodoridis, Georgios Arsenos

**Affiliations:** 1 Laboratory of Animal Husbandry, School of Veterinary Medicine, Faculty of Health Sciences, Aristotle University, 54124 Thessaloniki, Greece; 2 Clinic of Farm Animals, School of Veterinary Medicine, Faculty of Health Sciences, Aristotle University, 54124 Thessaloniki, Greece; 3 Laboratory of Animal Production Economics, School of Veterinary Medicine, Faculty of Health Sciences, Aristotle University, 54124 Thessaloniki, Greece

**Keywords:** dairy sheep, fertility, artificial insemination, housing conditions

## Abstract

**Simple Summary:**

Artificial insemination (AI) is a powerful tool for animals’ genetic improvement. However, its application in sheep remains relatively limited due to inconsistent results, since the success of the method is dependent on many factors. In our study, we investigated the impact of housing conditions during the summer period on the fertility of dairy sheep in Greece, after cervical AI with cooled semen. The pregnancy rate was found to be negatively affected by the ambient temperature and Temperature-Humidity Index inside the shed, space availability, frequency of bedding renewal, and outdoor access. Appropriate housing conditions could help to increase pregnancy rates following AI during the summer months.

**Abstract:**

The objective was to assess the effect of housing conditions during the summer months on the success rates of cervical artificial insemination (AI) with cooled semen, in intensively reared dairy ewes in Greece. The study involved 2083 Lacaune ewes from 23 flocks that were serviced during May to September. An estrous synchronization protocol with the insertion of progestogen sponges for 14 days and eCG administration at sponge removal, was used. All ewes were inseminated 54–57 h after sponge removal with cooled semen (15 °C) from 10 Lacaune rams. Pregnancy diagnosis was performed via trans-dermal ultrasonography at 35–40 days after AI. Data recording started the day after sponge placement (15 days prior to AI), and lasted up to 14 days after AI. Daily records included temperature, relative humidity, and Temperature-Humidity Index (THI) inside the shed. Available space and volume per animal, frequency of bedding renewal, access to a yard, and indoor light were also recorded in each farm. Binary logistic regression of data records showed that temperature and THI increases at days −15 to +4 around AI (day 0) had a negative effect on pregnancy rates (reducing the likelihood of pregnancy by 3–6% and 7%, respectively). The latter also decreased significantly (*p* < 0.05) in farms with high stocking density, non-frequent bedding renewal, and outdoor access by ewes (by 30%, 34%, and 44%, respectively). Overall, the results indicate that appropriate housing conditions are warranted to increase the success of AI in dairy ewes during the summer months.

## 1. Introduction

Dairy sheep production systems face diverse challenges in their efforts to secure overall sustainability, which in practice is dictated by the level of the ewe’s productivity [1]. The evidence in the literature suggests that in dairy flocks, efficiency, and enhanced productivity are directly related with reproductive success and the pace of genetic improvement [2]. The notion is that both can be materialized using assisted reproduction techniques focusing on cervical artificial insemination (AI), which is easy to perform and with relatively low cost [2,3]. AI has enhanced genetic improvement by increasing selection intensity and genetic evaluation precision [4,5]. However, the use of AI in dairy sheep, with the exception of France, is not as common as in other farm animals [6,7,8]. A major obstacle is the cervix anatomy of ewes, which allows only cervical insemination, preferably with cooled or chilled, but not with frozen-thawed semen [9,10].

Dairy sheep is a significant sector of livestock farming in Greece, and the national flock is ranked as the second largest in the EU [11]. Despite the fact that high producing breeds, such as Lacaune, are reared in Greece, overall productivity is rather low. The notion is that artificial insemination, combined with well designed genetic improvement programs, could help increase the milk production per ewe [12] and secure the sustainability of the sector [13]. However, the implementation of AI is not popular amongst sheep farmers because of inconsistent results. Previous studies have shown that factors associated with AI, including the health status of rams, as well as parity, lambing interval, and the body condition score (BCS) of ewes are key parameters of success, and therefore should be considered before AI implementation [8,14,15,16,17]. Moreover, in the light of climate change, outdoor temperature, relative humidity, and Temperature-Humidity index (THI) have been indicated as possible risk factors [18,19,20,21,22]. However, the role of indoor housing conditions on the success rates of AI in intensive dairy sheep farms has not been investigated, although research in dairy cows has shown a significant association between housing conditions and reproductive efficiency, AI success, and sexual behavior [2,23,24].

Therefore, our objective here was to investigate the effect of housing conditions and specific microclimatic variables during summer on the success of cervical AI with cooled semen in intensively reared dairy ewes in Greece.

## 2. Materials and Methods

### 2.1. Farms and Animals

The research reported here was conducted from May to September, over two consecutive years, 2018 and 2019. The study period was within the common breeding season for small ruminants in Greece, which is characterized by a high ambient temperature. A total of 2083 adult Lacaune ewes were used from 23 commercial flocks (36–122 ewes per flock) that practiced AI, located in Northern Greece. The flocks were selected based on willingness to perform AI and the ability to keep reliable records; inseminated ewes were selected by the farmers based on health and milk production records (the highest producing animals were chosen for AI). The ewes were 1–7 years old, and at the 5–7th month of their lactation; the average daily milk yield was 1.20 ± 0.34 L per ewe. The diet of the ewes was similar across the selected flocks; rations comprised alfalfa hay and alfalfa silage with a concentrate mix (0.8–1.2 kg/ewe/day, depending on actual milk production) of ground maize and barley grains, soybean meal, wheat middlings, and vitamin and mineral supplements. Wheat straw and water were available for ad libitum consumption. Table 1 shows some key characteristics of the studied farms.

### 2.2. Estrous Synchronization and AI Procedure

Estrus synchronization and AI in ewes were performed using the same protocols described by Priskas et al. [14]. Briefly, estrus synchronization was performed using intravaginal fluogestone acetate sponges (Chronogest CR, MSD Animal Health, The Netherlands) that were kept for 14 days. On the day of removal, each ewe was subjected to an intramuscular injection of 500 IU equine chorionic gonadotropin (Gonaser, Hipra Laboratorios, Girona, Spain). Semen was collected using an artificial vagina from 10 purebred Lacaune rams that were kept in an authorized semen collection center (OVIS PC, Thessaloniki, Greece). Each ejaculate was assessed for sperm concentration and mass motility based on the 0–5 scale suggested by Evans and Maxwell [25]. The ejaculates with concentration >3 × 10^9^ spermatozoa/mL and mass motility ≥4 were more processed. Then, the semen was diluted with skimmed milk to a final concentration of 1.6 × 10^9^ spermatozoa/mL [26], cooled at 15 °C, loaded into 0.25 mL mini-straws (IMV Technologies, L’Aigle, France), and transported to each farm at 15 °C [17]. Cervical fixed-time AI was performed 53–57 h after sponge removal and within eight hours after semen collection. In all farms, AI was performed by the same technician. Pregnancy diagnosis (PD) was performed 35–40 days after AI with ultrasonography, using a 5 MHz transducer with sector probe (Draminski Animal Profi, Szabruk, Poland).

### 2.3. Data Collection and Handling

A designated recording sheet was used for data collection. At the time of AI, ram semen identification was recorded for each ewe. Moreover, the Body Condition Score (BCS) of each ewe was assessed via palpation of the dorsal lumbar region and according to the 5-point scale (1—emaciated to 5—obese, with 0.25 increments) of Russel et al. [27]. The assessment was always performed by the same qualified veterinarian to eliminate classifier variability. BCS was transformed into a categorical variable with three classes: 1 = <2.25, 2 = ≥2.25, and <3.75, 3 = ≥3.75.

Housing conditions were evaluated on the basis of a designated protocol for measurements. Specifically, the length, width, and height of the pens were recorded, and the available space (m^2^) and volume (m^3^) per ewe were calculated. For each of the latter parameters, two categories were defined, space: 1 = <1.5 m^2^/animal, 2 = ≥1.5 m^2^/animal, according to Dwyer and Ruiz [28]; and volume: 1 = <9.5 m^3^/animal, 2 = ≥9.5 m^3^/animal, based on the median of the respective continuous variable.

Information on whether ewes had outdoor access to a yard was recorded as a binary variable. The frequency of bedding material renewal was also recorded, and the median of the variable was used to define two categories: 1 = ≤4 days, 2 = >4 days. Moreover, the level of natural light inside the shed was evaluated and scored as poor (dark areas inside the pen during the day), adequate (conditions similar to an overcast day), or good (ability to perform all visual tasks during the day). The latter was a subjective evaluation according to the evaluator’s experience.

In each farm, a portable data logger for temperature and relative humidity (LOG32TH, DOSTMANN Electronic GmbH, Wertheim-Reicholzheim, Germany) was placed in the middle of the shed where the ewes were kept, at 1.5 m above ground level, without contact to wall, and protected from direct sunlight. Temperature (°C) and relative humidity (%) were recorded every 10 min during the period of 15 days prior up to 14 days post-AI. The daily mean temperature and the mean relative humidity were calculated. The daily maximum and mean THIs (THI_max_ and THI_m_, respectively) for each studied day were calculated according to the following formulas suggested by Kelly and Bond [29], Finocchiaro et al. [30], and Ramon et al. [31].

(1) $${\mathrm{THI}}_{\max}{=T}_{\max}-\left[0.55\;\times \;\left(1-\frac{{\mathrm{RH}}_{\min}}{100}\right)\right]\;\times \;\left(T_{\max}-14.4\right)$$

(2) $${\mathrm{THI}}_{m}{=T}_{m}-\left[0.55\;\times \;\left(1-\frac{{\mathrm{RH}}_{m}}{100}\right)\right]\;\times \;\left(T_{m}-14.4\right)$$

where: T_max_ = daily maximum temperature (°C), T_m_ = daily mean temperature (°C), RH_min_ = daily minimum relative humidity (%), and RH_m_ = daily mean relative humidity (%).

Daily THI_max_, THI_m_, T_max_, and T_m_ at days −14, −7, −2, 0, +2, +7, and +14 around AI were used in the statistical analyses according to previous literature [15,16]. The averages of daily THI_max_, THI_m_, T_max_, and T_m_ were also calculated for the following periods around AI: −15 to −6, −5 to +4, and +5 to +14 days, to be used in the statistical analyses.

The observed frequencies for the categorical variables of housing conditions are summarized in Table 2. Descriptive statistics of all studied microclimatic variables for each of seven days around AI (−14, −7, −2, 0, +7, and +14 days) and the respective averages for three periods (−15 to −6, −5 to +4, and +5 to +14 days) are presented in Table 3 and Table 4, respectively. The final dataset used for the analysis is available in Dataset S1.

### 2.4. Statistical Analysis

Binary logistic regression analysis was performed using the R statistical package “stats” to estimate the effect of housing conditions on AI success. AI success was defined as a positive pregnancy diagnosis with ultrasonography, and was used as the dependent variable. Initially, the preliminary screening of all variables was performed for univariate associations; apart from the housing conditions (frequency of bedding renewal, available space and volume per ewe, outdoor access, level of natural light indoors, and microclimatic variables), ewe BCS, parity, and ram were also tested. The variables that were found to have a significant effect in the univariate analysis at a *p* < 0.20 level were included in a multivariate model; different models were constructed for each THI, temperature, and relative humidity variables, and for the frequency of bedding renewal. At the next stage, a stepwise regression approach was applied to build the final model in which all explanatory factors were significant at a 5% level. In each of the final multivariate models, the explanatory factors included ewe BCS, parity, available space per ewe, and outdoor access, as described below:

$$Y_{\mathrm{ijmn}}{=\mu +B}_{i}{\;+\;P}_{j}{\;+\;S}_{m}{\;+\;A}_{n}{+\;b1\times M+e}_{\mathrm{ijmn}}$$

where: Y_ijmn_ is AI success (0 = negative pregnancy diagnosis, 1 = positive pregnancy diagnosis), μ is the overall population mean, B_i_ is the fixed effect of ewe BCS (i = 2 levels, 1 = <2.25, 2 = ≥2.25, and <3.75, 3 = ≥3.75), P_j_ is the fixed effect of parity (j = 5 levels, 0 = yearlings, 1 = first parity, 2 = second parity, 3 = third parity, 4 = ≥fourth parity), S_m_ is the fixed effect of available space per ewe (m = 2 levels, 1 = <1.5 m^2^/animal, 2 = ≥1.5 m^2^/animal), A_n_ is the fixed effect of outdoor access (n = 2 levels, 0 = no access, 1 = access), b1 is the regression coefficient on each microclimatic variable M (temperature, relative humidity, THI), and e_ijmn_ is the residual error.

In all cases, a logit function for binomial distribution was assumed.

## 3. Results

**Figure 1:**
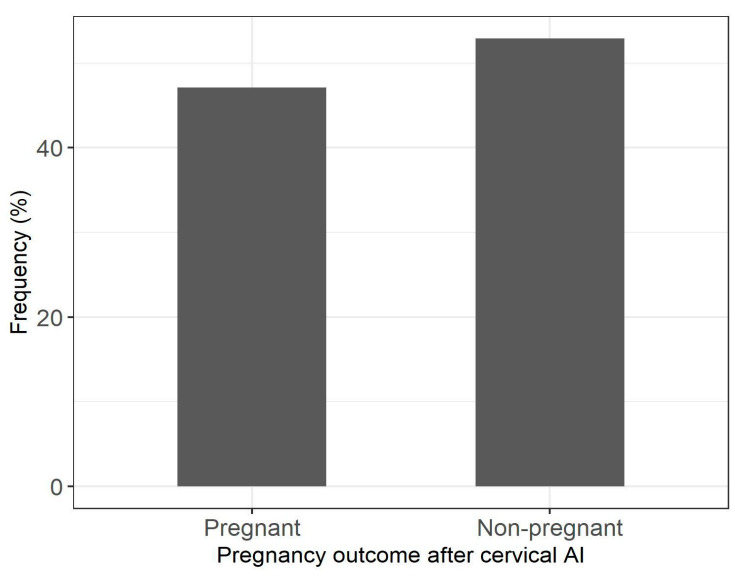
Pregnancy rate (%) following cervical AI with cooled semen (15 °C) in 2083 ewes.

Pregnancy rates following cervical AI in ewes are presented in Figure 1. Table 5 shows the results regarding the effects of housing conditions on AI success. Temperature and THI were significant (*p* < 0.05) risk factors. Specifically, for each unit increase in the daily maximum temperature at days −14, −7, −2, 0, and +2 around AI, the likelihood of pregnancy significantly decreased by 4–8%. Moreover, ewes were less likely to conceive for each unit increase in the average maximum temperature across days −15 to −6 and −5 to +4 (by 6 and 3%, respectively). Likewise, for each unit increase in the daily maximum THI at days −14, −7, −2, 0, and +2 around AI, the odds of pregnancy significantly decreased (3–8%); the odds were also decreased (by 7%) with the increase in the maximum THI values when averaged across days. Regarding the mean values of microclimatic variables, unfavorable associations with pregnancy rates were found for the mean temperature concerning days −14, −7, 0 and the period from day −15 to day −6 around AI (decrease by 5–9%), and for THI at days −15 to −6 and from day −5 to day +4 (decrease by 7% and 4%, respectively). In farms where access to a yard was not available, ewes were more likely to become pregnant (by 44%) following AI, compared to ewes with outdoor access. Moreover, the likelihood of pregnancy was higher by 30% and 34% when ewes had sufficient space available (>1.5 m^2^/animal) and their bedding was frequently renewed (≤4 days), respectively.

## 4. Discussion

As asserted in the introduction, the aim here was to assess the role of housing conditions on AI success. The latter is becoming a challenging issue for the future of sheep production in terms of the genetic improvement of flocks, and the economic viability and sustainability of farms. Considering the available literature, this is the first study addressing such a question, with a focus on microclimatic variables in intensively reared dairy ewes. The results showed that indoor temperature and THI from −15 to 4 days around AI, space allowance, the frequency of bedding renewal, and outdoor access play a significant role in pregnancy rates. Previous studies in sheep have focused on estimating the effect of outdoor environmental conditions on AI success. However, the evidence from research in dairy cows suggests that outdoor environmental variables differ significantly from those indoors and subject to different designs of buildings and available equipment [32]. Such variations lead to an underestimation of heat stress and its consequences in animal performance. Therefore, in the present study, microclimatic parameters in different farms were recorded to evaluate their association with AI success in intensively reared dairy ewes. According to Anel et al. [17], season is also a significant factor affecting the success of AI; pregnancy rates, following AI in the summer months, are considerably lower compared to the rest of the year. Hence, we deliberately chose to focus the present study in the most challenging period from May to September.

The work of Santolaria et al. [19], has shown that pregnancy rates in Rasa Aragonesa were significantly reduced when the average outdoor temperature was above 30 °C two days prior to AI. Moreover, Palacios et al. [18] reported a negative association between the daily maximum temperature on the day of AI and the pregnancy rates of Churra dairy ewes in Spain. Those findings are in accordance with the present study regarding indoor daily maximum temperature. Moreover, the present study also showed significant effects of high temperatures during a wider range of days around AI (−15 to +4 days). In particular, the mean temperature at days −15 to −6 seems to be a significant risk factor, which has not been revealed in other studies. Specifically, Santolaria et al. [19], reported no significant effects of temperature at days −12 to 0, −2 to 0, 0 to +2, and 0 to +14 on pregnancy rates following AI. Our results provide novel evidence based on the continuous recording of indoor temperature in different farms that is indicative of the dominant role of microclimatic conditions on AI success, compared to studies with outdoor records.

The results showed that mean relative humidity was not a significant risk factor in the success rates of AI. This is in agreement with previous studies in Spanish flocks [18,19]. The THI, which combines the data of temperature and relative humidity, is considered to be a reliable indicator of heat stress [33]. In the present study, the increase in the daily THI_max_ and THI_m_ over a wide range of days (−15 to +4) around AI was found to adversely affect pregnancy rates. Similar findings, but only for the day of AI, were reported by two studies conducted in Spain. Palacios et al. [18] showed that during summer, the lower the THI on the day of AI, the higher the fertility of dairy ewes. Abecia et al. [20] reported that Rasa Aragonesa ewes under severe heat stress had lower fertility rates. However, in the study of Santolaria et al. [19], there were no significant differences concerning THI_max_ and THI_m_ during the period of −12 to +14 days around AI. In studies with dairy goats, THI_m_ on the day of AI was a risk factor for AI success rate in goats of Murciano-Granadina breed, but no significant differences were observed in goats of Florida breed [19,34]. Such discrepancies are most likely due to differences in experimental designs such as sample size, farming system, nutritional management, and breeds of animals. In the present study, indoor temperature and THI within the period −15 days to +4 days regarding the time of AI, are both significant factors for AI success. This is in line with a recent study suggesting that ewe reproductive performance is mostly greatly affected by heat stress one week before and until 5 days after estrus [22]. Our results are in agreement with similar studies in dairy cows that confirmed that THI is a very important climatic factor for AI success [35,36,37,38]. The evidence from studies in dairy cows suggests that heat stress could compromise follicular growth, selection, and dominance, thus affecting the quality of ovarian follicles and ovulation [39,40]. Ιn Merino sheep, thermal stress can result in a later estrus of shorter duration [41]. Regarding the period post-AI, adverse effects of the increase in temperature and THI on AI might be related to embryo susceptibility. Specifically, Gharibzadeh et al. [42] suggested that heat stress in ewes can impact oocyte maturation, therefore affecting embryonic development. This effect seems to be limited to the first 8 days after AI, which corresponds to the susceptible period of early embryonic development and the embryo’s entrance to the uterus [43]. After this period, embryonic resistance to heat stress is increased [44].

According to the present findings, emphasis should also be given on improving other housing conditions related to management practices in order to increase the success rates of AI. Space allowance (the average area available per ewe) is considered an important welfare indicator, especially in intensive farming systems, and a minimum available area of 1.5 m^2^/ewe is strongly recommended. Previous research in dairy sheep has shown that a limited area per ewe (<1.5 m^2^) is linked to a reduced immunity response and milk production [33,45,46,47]. However, there is no available literature regarding the role of this factor on AI success in dairy ewes. Our study provides novel evidence that inadequate space allowance is associated with decreased AI success rates; ewes under such conditions were around 1.3 times less likely to conceive. Similar findings stemmed from research in dairy cows showing that inadequate space per animal was linked to reduced pregnancy rates [23,48]. Moreover, animals reared under such housing conditions are subjected to high stress, expressing aggressive behavior due to reduced lying time, and limited access to feed and water. All the above can lead to insufficient feed intakes, with adverse effects on fertility [28,49]. Therefore, such stressful factors could possibly explain the reduced success of AI reported in our study when the available area per ewe was limited (<1.5 m^2^). On the other hand, the present study indicated no significant effects of available volume per animal on AI success. Sevi et al. [50] set a cut-off point on the sufficient airspace allowance for sheep at 7 m^3^/animal; below this threshold, negative effects on productivity and udder health have been reported. In our study, all ewes were allowed a volume of more than 7 m^3^/animal (ranging from 8 to 13 m^3^); hence, a higher threshold at 9.5 m^3^/animal was set to study the association with AI success. Under such conditions of sufficient airspace, the results suggested that no adverse effects on AI success are expected. However, future research should further investigate the effect of available volume per animal on pregnancy rates following AI by comparing sufficient and insufficient airspace allowance conditions.

In intensive sheep farming systems, inappropriate litter management and non-frequent bedding renewal have been shown to compromise animal welfare [33,51]. Although there is no available literature on the possible effects on the success rates of AI, poor animal welfare could potentially explain the reduced pregnancy rates reported in our study when bedding was not frequently renewed (>4 days). Moreover, infrequent bedding renewal leads to manure and urine accumulation, which favors microorganism development, thus increasing disease incidence [51]. In this regard, a predisposition to health issues may further explain the reduced success of AI in the case of the infrequent bedding renewal reported in our study. This is further supported by the study of Caraviello et al. [48] that suggested the reduced reproductive performance of dairy cows due to health issues such as mastitis as a result of the inappropriate management of organic bedding materials.

Access to an outdoor space is considered important for the welfare of intensively reared dairy ewes, since it reduces stereotypic behaviors [46,52]. However, in our study, ewes that had access to a yard around AI days were less likely to have a positive pregnancy outcome. This could be potentially attributed to increased locomotion activity and a higher energy demand for the thermoregulation of ewes when outdoors, compared to animals that remained inside the pen [52]. Increased activity might be influencing semen flow towards the uterus and/or impacting on embryo implantation in the days after AI. Nevertheless, to support this statement, a study focusing on the effect of locomotion activity on AI success is needed.

Photoperiod plays an important role on reproductive seasonality and other biological functions of sheep [33,53]. However, Boivin [54] reported no significant associations between reproductive efficiency and light intensity in dairy sheep farms. This is in accordance with our results, where the level of natural light in the shed was not found to significantly affect AI success. It should be noted that the percentage of studied farms with poor natural light was very low (4%), and this could have led to an underestimation of the relevant association. Therefore, future studies with a more balanced sample size concerning the relative housing parameter are warranted.

Overall, based on our findings, appropriate housing conditions are an issue of substantial importance for increasing the success of AI in intensively reared dairy ewes during the summer months. This importance is expected to be even greater in the near future, in the light of climate change. Moreover, increasing the success rates of AI, and hence, its overall acceptability by dairy sheep farmers, is expected to be beneficial for the economic performance of farms. In this regard, appropriate management practices for decreasing indoor temperature and THI, such as natural and mechanical ventilation, available space of greater than 1.5 m^2^/ewe, a frequency of bedding renewal of less than four days, and limited outdoor access of animals at the period around AI, are suggested in order to improve fertility rates.

## 5. Conclusions

Considering the results of the present study, indoor temperature and THI, space allowance, frequency of bedding renewal, and outdoor access of ewes to a yard are key factors of success of AI in intensively reared dairy ewes in Greece. During the summer months, appropriate measures to reduce animal heat stress, adequate space availability, the frequent renewal of bedding material, and the limited outdoor access of inseminated ewes could help to increase pregnancy rates following AI, and hence improve the popularity for its implementation. The latter would be beneficial in terms of production and the overall sustainability of intensive dairy sheep farming systems.

## Figures and Tables

**Table 1:** Key characteristics of dairy sheep farms used in the study.

Characteristics of Selected Flocks	Mean (SD ^1^)
Average number of adult ewes	512.6 (386.56)
Average number of rams	20.3 (13.41)
Age of yearlings at first mating (months)	8.8 (1.45)
Milk production (kg/ewe/lactation period) in liters	361.7 (52.63)

^1^ SD = standard deviation.

**Table 2:** Frequency (%) and corresponding number of observations in parenthesis, of the studied housing conditions.

Variable	Category	Frequency
Access to yard	No	65.4 (1362)
	Yes	34.6 (721)
Space per animal	<1.5 m^2^/animal	29.9 (623)
	≥1.5 m^2^/animal	70.1 (1460)
Volume per animal	<9.5 m^3^/animal	55.2 (1149)
	≥9.5 m^3^/animal	44.8 (934)
Bedding renewal	≤4 days	61.8 (1287)
	>4 days	32.8 (796)
Indoor light	Poor	4.0 (83)
	Adequate	58.3 (1215)
	Good	37.7 (785)

**Table 3:** Descriptive statistics (mean ± SD in parenthesis) of microclimatic variables measured in the studied farms in relation to the days of artificial insemination.

	Days in Relation to AI						
Variable	−14	−7	−2	0	+2	+7	+14
T_m_^1^ (°C)	22.1 (2.75)	22.9 (3.02)	23.1 (3.97)	22.6 (4.07)	22.5 (4.68)	22.3 (4.68)	22.8 (5.09)
T_max_^2^ (°C)	29.4 (3.28)	31.0 (3.32)	30.7 (2.97)	30.4 (4.34)	29.9 (5.09)	30.0 (5.47)	29.9 (6.55)
RH_m_^3^	0.6 (0.07)	0.7 (0.05)	0.6 (0.08)	0.6 (0.07)	0.6 (0.08)	0.6 (0.06)	0.6 (0.13)
THI_m_^4^	20.3 (2.28)	20.9 (2.46)	21.2 (3.28)	20.6 (3.20)	20.5 (3.68)	20.3 (3.67)	20.7 (3.88)
THI_max_^5^	24.5 (2.30)	25.6 (2.39)	25.4 (3.13)	25.3 (3.04)	24.9 (3.32)	24.2 (5.0 3)	25.0 (4.02)

^1^ T_m_ = daily mean temperature, ^2^ T_max_ = daily maximum temperature, ^3^ RH_m_ = daily mean relative humidity, ^4^ THI_m_ = daily mean temperature-humidity index, ^5^ THI_max_ = daily maximum temperature-humidity index.

**Table 4:** Descriptive statistics (mean and standard deviation in parenthesis) of the average of microclimatic variables measured in the studied farms for three periods around artificial insemination.

	Periods Around AI (days)		
Variable	−15 to −6	−5 to +4	+5 to +14
T_m_ ^1^ (°C)	22.5 (2.55)	21.7 (5.70)	22.9 (5.13)
T_max_^2^(°C)	30.2 (2.80)	30.3 (4.07)	30.0 (5.64)
RH_m_^3^	0.6 (0.06)	0.6 (0.06)	0.6 (0.07)
THI_m_^4^	20.7 (2.17)	20.8 (2.87)	20.5 (3.79)
THI_max_^5^	24.9 (2.19)	24.9 (2.89)	24.8 (3.76)

^1^ Tm = daily mean temperature, ^2^ Tmax = daily maximum temperature, ^3^ RHm = daily mean relative humidity, ^4^ THIm = daily mean THI, ^5^ THImax = daily maximum THI.

**Table 5:** Results of odds ratios (95% confidence intervals) of housing conditions on artificial insemination success.

	Categories			95% CI		
Variable	Compared	Reference	Odds Ratio	Lower	Upper	*p*-Value
Access to yard	No	Yes	1.47	1.21	1.78	<0.001
Space	≥1.5 m^2^/animal	<1.5 m^2^/animal	1.30	1.06	1.57	0.009
Bedding renewal	≤4 days	>4 days	1.34	1.11	1.61	0.002
T_max_^1^ (d ^5^ −14)	Continuous		0.96	0.93	0.98	0.005
T_max_^1^ (d ^5^ −7)	Continuous		0.95	0.92	0.97	<0.001
T_max_^1^ (d ^5^ −2)	Continuous		0.97	0.95	0.99	0.013
T_max_^1^ (d ^5^ 0)	Continuous		0.97	0.95	0.99	0.020
T_max_^1^ (d ^5^ +2)	Continuous		0.98	0.96	0.99	0.049
T_max_^1^ (d ^5^ −15 to −6)	Continuous		0.94	0.90	0.96	<0.001
T_max_^1^ (d ^5^ −5 to +4)	Continuous		0.97	0.95	0.99	0.033
T_m_^2^ (d ^5^ −14)	Continuous		0.96	0.93	0.99	0.029
T_m_^2^ (d ^5^ −7)	Continuous		0.95	0.92	0.98	0.002
T_m_^2^ (d ^5^ 0)	Continuous		0.97	0.95	0.99	0.050
T_m_^2^ (d ^5^ −15 to −6)	Continuous		0.95	0.91	0.99	0.021
THI_max_^3^ (d ^5^ −14)	Continuous		0.95	0.91	0.99	0.008
THI_max_^3^ (d^5^ −7)	Continuous		0.92	0.89	0.96	<0.001
THI_max_^3^ (d^5^ −2)	Continuous		0.94	0.91	0.97	0.001
THI_max_^3^ (d ^5^ 0)	Continuous		0.96	0.93	0.99	0.01
THI_max_^3^ (d ^5^ +2)	Continuous		0.97	0.94	0.99	0.026
THI_max_^3^ (d ^5^ −15 to −6)	Continuous		0.93	0.89	0.97	0.002
THI_m_^4^ (d ^5^ −15 to −6)	Continuous		0.93	0.89	0.97	0.002
THI_m_^4^ (d ^5^ −5 to +4)	Continuous		0.96	0.93	0.99	0.020

^1^ T_max_ = daily maximum temperature, ^2^ T_m_ = daily mean temperature, ^3^ THI_max_ = daily maximum THI, ^4^ THI_m_ = daily mean THI, ^5^ d = days around artificial insemination.

## Data Availability

Data presented in this study are contained within the article and Supplementary Material (Dataset S1).

## Supplementary Materials

The following supporting information can be downloaded at: https://www.mdpi.com/article/10.3390/ani12192693/s1. Dataset S1: Total dataset of traits used for the analyses.
